# Design and Characterization of Hybrid Gelatin/PEGDA Hydrogels with Tunable Viscoelastic Properties

**DOI:** 10.1021/acs.biomac.5c01048

**Published:** 2025-07-25

**Authors:** Pietro Renato Avallone, Nadia Russo, Nicola Gargiulo, Nino Grizzuti, Salvatore Costanzo

**Affiliations:** † Department of Chemical, Materials, and Production Engineering, 9307 Federico II University, P.le Tecchio 80, Naples 80125, Italy; ‡ Center of Advanced Measurement and Technology Services (CeSMA), 9307 Federico II University, Corso N. Protopisani, Naples 80146, Italy

## Abstract

We report on the formulation and characterization of hybrid hydrogels composed of gelatin and poly­(ethylene glycol) diacrylate (PEGDA). Such hydrogels undergo sol–gel transitions either reversibly via temperature variation or irreversibly via UV photopolymerization. By finely tuning the interplay between physical (thermal) and chemical (UV-induced) gelation mechanisms, a broad spectrum of viscoelastic properties and swelling ratios can be achieved. We systematically investigate the effects of PEGDA concentrations and the preparation protocol on gelation kinetics and the mechanical properties, morphology, and swelling of the resulting hydrogels. Rheological measurements demonstrate that a higher gelatin content promotes faster physical gelation and enhances the elastic properties, while UV-triggered PEGDA cross-linking competes with and modifies the physical network, especially at elevated PEGDA levels. SEM analysis reveals that increasing the level of PEGDA leads to denser microstructures with reduced porosity. Swelling tests indicate that lower PEGDA concentrations result in greater water uptake. Our findings highlight the synergistic interactions between reversible and irreversible cross-linking mechanisms and their role in modulating the final hydrogel properties. The tunability of this system offers promising potential for applications that require customizable mechanical behavior and morphological characteristics.

## Graphical abstract

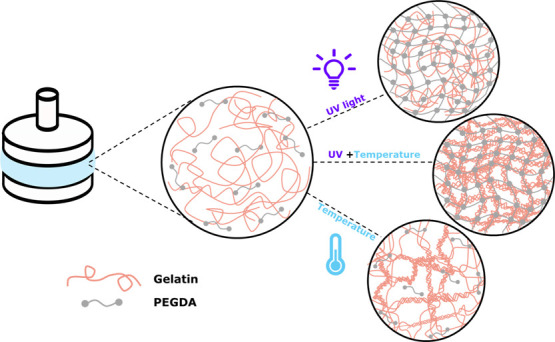

## 1 Introduction

Gelation, also known as the sol–gel transition, is the process by which a polymeric or colloidal solution transforms into a soft solid.^1^ On the microscopic scale, this transition involves the formation of a 3D network resulting from associations between polymers or particles. The reversibility of this transition depends on the nature of the cross-linking: if the cross-links are permanent covalent bonds, the transition is irreversible. On the contrary, it remains reversible when determined by physical interactions such as hydrogen bonding, electrostatic forces, or van der Waals attractions.^2^

Biopolymers are a well-known class of materials that can form physical gels when dissolved in water.^3,4^ They can derive from animal sources, seaweeds, and trees or can be produced through fermentation processes.^5^

One of the primary mechanisms of hydrocolloid gelation is cold-set gelation. It is a process in which gel formation occurs as biopolymer solutions are cooled. Typically, these solutions are prepared by dissolving hydrocolloid powders in a hot solvent, most commonly water. Upon cooling, the polymer chains become enthalpically stabilized, leading to the development of a structured network that forms the gel.^6^ Common biopolymers that undergo cold-set gelation include gelatin, agar, and κ-carrageenan.^7,8^

Gelatin is a protein mainly derived from animal byproducts such as pig skin, bovine and porcine cartilage, bones, and hides, through the partial hydrolysis of collagen.^9^ It is used across various sectors, including food, cosmetic, medical, and pharmaceutical industries.^10–12^ Its thermoresponsive behavior and favorable biocompatibility profile, which can be tailored according to the intended biological application and implantation site, make gelatin particularly attractive for biomedical applications, including tissue engineering scaffolds, drug delivery matrices, and wound healing constructs.^13–15^ Physically cross-linked gelatin gels often have limited mechanical stability and temperature sensitivity, which can restrict their long-term functionality in physiological environments, particularly when structural integrity under mechanical loading, such as in cartilage regeneration or vascular grafting, is essential.^16–19^ To overcome these limitations, gelatin is frequently combined with synthetic polymers such as poly­(ethylene glycol) (PEG) to create hybrid hydrogels with enhanced mechanical and structural properties.^20,21^

PEG is a synthetic, hydrophilic polymer widely used in biophysical and biomedical applications, such as hydrogel-based drug delivery systems, tissue-engineered scaffolds, and cell encapsulation platforms, due to its chemical versatility, tunable biocompatibility dependent on the biological context, and favorable permeability characteristics toward essential gases and nutrients.^22–24^ Its basic structure, composed of repeating ethylene oxide units and terminal hydroxyl groups, can be functionalized with a variety of reactive groups—such as amines, carboxyls, thiols, and acrylates—enabling covalent cross-linking and incorporation of bioactive moieties.^25^ Among these derivatives, poly­(ethylene glycol) diacrylate (PEGDA) has gained significant attention for its ability to form hydrogels via free radical photopolymerization in the presence of a photoinitiator.^26^

Photopolymerization offers several advantages, including rapid gelation, precise spatiotemporal control via light exposure, low heat generation and temperature sensitivity, and the ability to modulate mechanical properties by tuning light intensity and exposure time.^27^ This process allows the formation of robust three-dimensional networks under mild conditions, compatible with the encapsulation of cells and bioactive molecules.

PEGDA-based hydrogels, especially when combined with natural biopolymers like gelatin, provide a favorable environment for cell proliferation and tissue regeneration.^28^ Their high water content and structural similarity to the extracellular matrix (ECM) support a hydrated, cell-friendly microenvironment.^29^ Such hybrid systems are particularly well-suited for applications in wound healing, drug delivery, and tissue engineering.^30–32^

Recent investigations have demonstrated the potential of gelatin as a co-initiator in the UV-induced cross-linking of PEGDA-based hydrogels.^33–38^ Moreover, PEGDA–gelatin bioinks are increasingly attracting interest within the scientific community due to their promising applicability in advanced biofabrication strategies. However, a systematic investigation about the influence of temperature and UV exposure on the gelation kinetics and the mechanical properties of the resulting hydrogels is currently lacking.

In the above context, rheology provides a powerful tool for monitoring the sol–gel transition and measuring the viscoelastic properties of hydrogels under different stimuli.^39,40^ Ad hoc rheological setups enable precise control over both light exposure and thermal history during time-dependent gelation tests. This approach allows for a fine modulation of the gelation kinetics and real-time monitoring of the evolution of mechanical properties.

The aim of this study is to provide a comprehensive investigation of the gelation kinetics, encompassing both physical and chemical cross-linking mechanisms, of hybrid hydrogels composed of gelatin and PEGDA. Rheological measurements were employed to monitor, in situ, the gelation process and to evaluate the interplay between physical and chemical gelation mechanisms. In addition, scanning electron microscopy (SEM) was utilized to identify the morphology of the hydrogels, providing insights into their microstructure, and swelling measurements were performed to assess the water uptake capacity of the hydrogels, an important factor for applications. Furthermore, helium pycnometry was applied to determine the skeletal density of the hydrogels, offering valuable data regarding their compactness and structural integrity. By combining these analytical techniques, we aim to provide a comprehensive characterization of the hybrid hydrogel systems, their gelation kinetics, and their potential for applications such as biomedical and 3D printing.

## 2 Materials and Methods

### 2.1 Materials

Gelatin from porcine skin (gel strength 300, type A), poly­(ethylene glycol) diacrylate (PEGDA) (*M*_*n*_ = 700 g/mol), and 2-hydroxy-4-(2-hydroxyethoxy)-2-methylpropiophenone, commonly known as Irgacure 2959 (I2959), were purchased from Sigma-Aldrich and used without additional treatment.

#### 2.1.1 Preparation of the PEGDA/Water Solutions

Two different aqueous solutions were prepared, containing PEGDA at concentrations of 5 and 10 wt % (henceforth referred to as P5 and P10, respectively). Photoinitiator I2959 was added to each solution at a concentration of 1 wt % (see Table 1). PEGDA and I2959 were dissolved in bidistilled water in glass vials at room temperature. The mixtures were subjected to magnetic stirring at 360 rpm for 3 h to ensure complete dissolution. To prevent photopolymerization triggering due to light exposure, the vials were wrapped in aluminum foil.

**Table 1:** Composition of the Prepared Solutions

sample	gelatin/water	gelatin [wt %]	PEGDA [wt %]	I2959 [wt %]
P5			5	1
P10			10	1
G2	2/98	2		
G2-P1	2/98	2	1	1
G6	6/94	6		
G6-P1	6/94	6	1	1
G6-P5	6/94	5.7	5	1
G6-P10	6/94	5.3	10	1

#### 2.1.2 Preparation of the Gelatin/Water Solutions

Two gelatin/water solutions were prepared, containing gelatin at concentrations of 2 and 6 wt %, hereafter referred to as G2 and G6, respectively (see Table 1). Gelatin powder was dissolved in bidistilled water, and the resulting mixtures were magnetically stirred at 360 rpm and 60 °C for 30 min to ensure complete dissolution. The selected concentrations were chosen such that G2 is in the dilute regime, while G6 falls within the semidilute regime.^39^

#### 2.1.3 Preparation of the Hybrid Prepolymerized Solutions

PEGDA and I2959 were added to 10 g of the aqueous animal gelatin solution to obtain hybrid solutions with PEGDA concentrations of 1, 5, and 10 wt % while keeping the photoinitiator concentration at 1 wt %. Such a protocol ensures that the hybrid solutions have the same gelatin-to-water weight ratio as the pure gelatin-water solutions.

Each solution was mixed on a magnetic stirrer at 360 rpm and 60 °C for 24 h and subsequently stored in a refrigerator at 5 °C. To prevent light exposure, the glass bottles were wrapped in aluminum foil. The prepared hybrid samples are listed in Table 1.

### 2.2 Rheological Tests

Rheological tests were performed using an MCR702 rotational rheometer (Anton Paar, Austria) in a single-motor configuration with parallel plates (25 mm stainless-steel upper plate and quartz bottom plate) at a fixed gap of 1 mm. Temperature control was provided by a Peltier unit (plate and hood H-PTD200), and UV curing was achieved with a portable UV lamp (254/365 nm, 0.40 mW/cm^2^) positioned at a distance of 13 cm below the bottom plate.^41^ To prevent both sample evaporation and UV-light-driven oxidation, a low-viscosity silicone oil (η = 0.1 Pa·s at ambient temperature) was poured around the rim of the upper plate. The hood also served to confine UV light.

Two types of experiments were carried out:

- Dynamic temperature ramp tests (DTRTs) were performed at a frequency ω = 10 rad/s and strain γ = 5%. Such a strain value falls within the linear viscoelastic regime. While imposing oscillatory strain, the temperature was varied with a fixed rate of 3 °C/min. The samples were loaded at 60 °C, cooled to −5 °C and, after a waiting time of 300 s at −5 °C, heated again to 60 °C. With such a procedure, during physical gelation, the curves of the complex modulus feature a hysteresis between cooling and heating (Figure 1b). The transition temperatures were defined as the minimum of the derivative of log­(|G*|) with respect to temperature.^42,43^ The transition temperature during cooling is indicated as *T*_sol–gel_, whereas the transition temperature during heating is indicated as *T*_gel–sol_.
- Dynamic time sweep tests (DTSTs) were conducted at fixed temperature at γ = 5% and ω = 10 rad/s. We followed the protocol illustrated in Figure 1: the samples were initially loaded at 60 °C (*T*_0_) and then cooled at a rate of 3 °C/min to a selected target temperature *T*_1_ on the cooling curve of the complex modulus (colored diamonds in Figure 1b). Then, the ramp was stopped, and the viscoelastic moduli were measured over a time interval Δ*t*. The isothermal tests were performed with and without UV irradiation, depending on the investigated gelation mechanism. The gel time, *t*_*g*_, was defined as the crossover point between the storage modulus and the loss modulus.^44^

**Figure 1:**
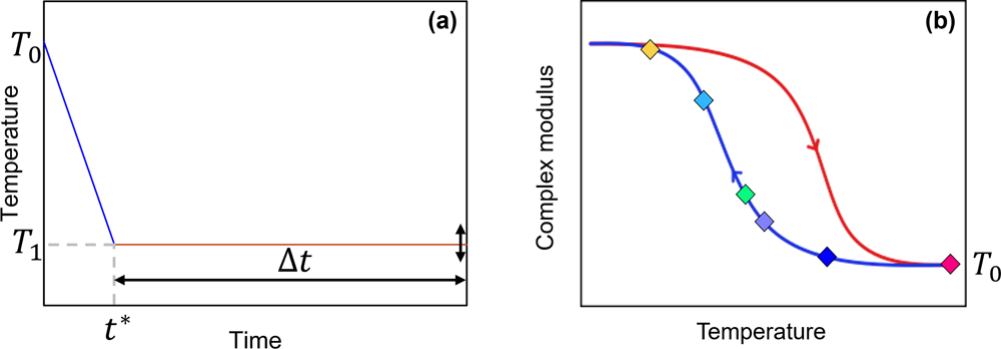
Qualitative sketches of (a) thermal history applied to the samples during a DTST experiment, and (b) temperatures, *T*_1_, selected from the hysteresis loop formed during the cooling and heating ramps of the physical gels.

### 2.3 Swelling Analysis and Gel Fraction Determination

To determine the water swelling behavior of the cross-linked samples, the hydrogels formed in the rheometer were carefully removed from the measuring geometry and then dried in a vacuum oven (Colaver M40-VT) at 40 °C for 24 h and weighted afterward. After that, they were put in a vial containing 10 mL of bidistilled water and stored for 48 h in an incubator at 25 °C. During this period, the samples were removed from the vial every 24 h, blotted to remove excess water, and weighed. The weight sampling at 24 and 48 h yielded virtually identical values, indicating that equilibrium was already achieved after 24 h. The water uptake capacity is expressed by the weight swelling ratio defined by eq 1:

$$\mathrm{Swelling}\;\mathrm{ratio}(\%)=\frac{W_{s}}{W_{d}}\times 100$$ 1

where *W*_*s*_ and *W*_*d*_ are the weight of the swollen and dried gel, respectively.^45^

After reaching the water swelling equilibrium, the hydrogels were dried again in a vacuum oven. The gel fraction was calculated by the following eq 2:^36^

$$\mathrm{Gel}\;\mathrm{fraction}(\%)=\frac{W_{g}}{W_{d}}\times 100$$ 2

where *W*_*g*_ is the dried weight of the sample after extraction of soluble parts.

### 2.4 SEM Analysis

Samples for SEM analysis were stored at −18 °C for 24 h and then freeze-dried using a lab-scale freeze-dryer (Christ Alpha 1–2 LDplus, Martin Christ, Osterode am Harz, Germany). Freeze-drying lasted 24 h, consisting of a main drying phase at a chamber temperature of −20 °C, a pressure of 10^2^ Pa for 12 h, and a final drying phase for the removal of the residual moisture at a temperature of −56.5 °C and a pressure of 1.7 Pa for 12 h.^46^

SEM analysis was performed by means of a Tescan Vega 4 instrument equipped with both secondary electron (SE) and backscattered electron (BSE) detectors. The employed electron landing energy was 25 keV. The working distance (WD) was kept as low as allowed by the height of the mounted samples. The latter approach, together with low beam currents (≤30 pA), allowed us to obtain the best possible imaging resolution. Because of their nonconductive nature, the samples were preliminarily gold-sputtered using a Cressington 108 coater.

### 2.5 Helium Pycnometry

Skeletal densities of the freeze-dried samples were calculated by using a Micromeritics AccuPyc II 1345 helium pycnometer equipped with a 1 cm^3^ nominal volume cell. Tests were carried out at ambient temperature, imposing a testing pressure of 152 kPa. Because of its hydrophilicity, every sample was preliminarily purged 30 times to avoid moisture outgassing during the analysis.

## 3 Results and Discussion

### 3.1 Thermally Driven Physical Gelation

Since PEGDA forms a network only under UV radiation, the gelation of aqueous PEGDA/gelatin solutions upon cooling and without UV application is primarily governed by gelatin. However, the question arises whether PEGDA can synergistically contribute to thermal gelation.

As an example, Figure 2a illustrates the evolution of the viscoelastic moduli as functions of temperature for the G6-P5 sample during a DTRT. At 60 °C, the viscoelastic moduli are low, and the system has Newtonian behavior (see Figure S2 of the Supporting Information). During the cooling phase, the viscoelastic moduli (blue symbols) exhibit an abrupt increase as the temperature approaches approximately 18 °C, corresponding to the onset of gelation.^7^ The onset temperature marks the formation of triple helices typical of the sol–gel transition of gelatin. At lower temperatures, when gelation is complete, the system exhibits solid-like behavior (see Figures S2 and S4 of the Supporting Information), with the storage modulus (*G*′) significantly higher than the viscous modulus (*G*″). At this stage, the triple helices are well-connected and form a 3D network. Conversely, during the heating phase (red symbols), the moduli decrease as the temperature rises. The reversibility of the transition is evidenced by the recovery of both *G*′ and *G*″ to their initial values at 60 °C.^47^

**Figure 2:**
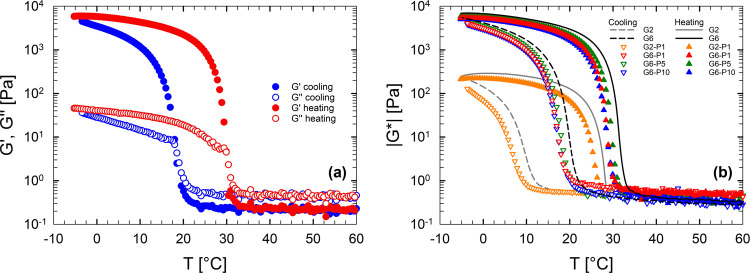
(a) Storage modulus *G*′ and loss modulus *G*″ as functions of the temperature for sample G6-P5 at 3 °C/min. Blue symbols represent the cooling phase, red symbols, the heating phase. (b) Complex modulus as a function of temperature at 3 °C/min. Down-pointing triangle symbols indicate the cooling phase, up-pointing triangle symbols, the heating phase for the hybrid hydrogels. The dashed and solid lines correspond to the cooling and heating curves, respectively, for the pure gelatin/water solutions G2 (gray lines) and G6 (black lines).

Figure 2b illustrates the thermal evolution of the complex modulus, |G*|, for all of the examined samples. For comparison, the sol–gel transition of a pure gelatin/water solution at the same gelatin/water ratio as the hybrid systems is also included. For the three samples with a 6/94 gelatin-to-water weight ratio, the hysteresis is nearly identical, irrespective of the PEGDA concentration. The curves of the G2-P1 solution differ from those of the other samples due to its lower gelatin concentration and different dilution regime.

The addition of PEGDA to the solution has a minimal effect on the elastic modulus of the final gel, indicating that the formed 3D network is dominated by the cross-links formed by gelatin. The gelation and melting temperatures of pure gelatin/water solutions are only slightly higher than those of the hybrid solutions. This suggests that the addition of PEGDA slows down the nonisothermal gelation kinetics. Such an effect can be explained in two ways. On the one hand, the presence of PEGDA may limit water availability for gelatin swelling. On the other hand, the formation of PEGDA/gelatin hydrogen bonds may hinder the formation of the triple helices that generate the gelatin network. In all cases, however, the viscoelastic moduli of the resulting gels are nearly identical, indicating that the structure of the final gel is not affected by PEGDA, although the inclusion of relatively small molecules in the core of the triple helices is also possible.^42^ Another interesting aspect is that at moderate concentrations (from 1 to 10 wt %), PEGDA concentration has a negligible effect on the transition temperatures, as the data of G6-P1, G6-P5, and G6-P10 nearly overlap.

### 3.2 Physical Gelation under Isothermal Conditions

To study the physical gelation of the samples under isothermal conditions, the protocol in Figure 1a was adopted. Each sample was loaded and thermally equilibrated at 60 °C, then it was cooled down to a target temperature, *T*_1_, at a fixed cooling rate (3 °C/min), and the viscoelastic moduli were measured as functions of time. The UV light was kept off for the entire duration of the experiment. As an example, Figure 3 presents the time evolution of G*′* and G^*″*^ for the samples G6-P5 at different target temperatures.

**Figure 3:**
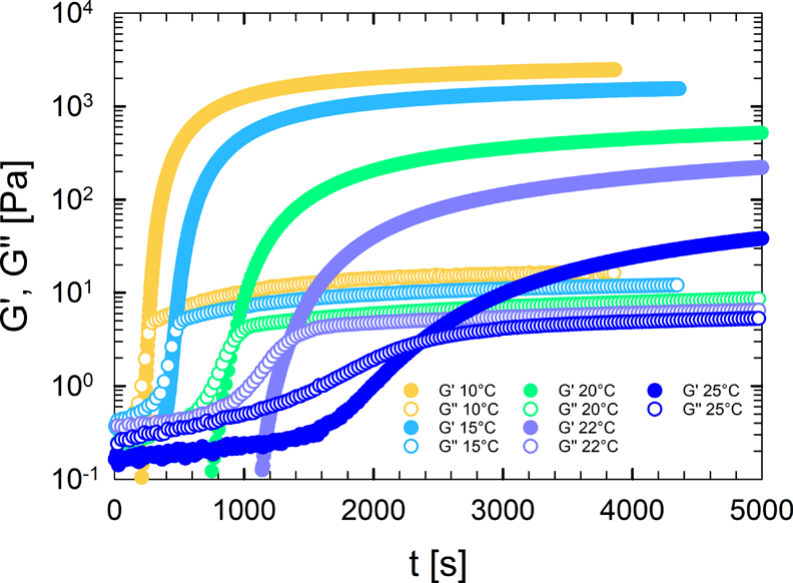
Storage modulus *G*′ and loss modulus *G*″ as functions of time for samples G6-P5 at selected temperatures.

At the selected temperatures, the system is initially liquid, with *G*″ ≫ *G′*. After an induction period, the duration of which depends on the target temperature, both moduli exhibit a significant increase that indicates the development of a gel structure. At a characteristic time, *t*_*g*_, a crossover occurs between *G*′ and *G*″. The time *t*_*g*_ is considered the gelation time of the sample^44^ and is reproducible with an error of less than 5%. As expected, the gelation process is faster when the isothermal test is conducted at temperatures closer to the gelation point, resulting in a decrease in the gelation time with decreasing temperature. Moreover, the long-time value of *G*′ of the gelatin-based hydrogels increases with temperature and does not reach a plateau, a characteristic fingerprint of the gelation kinetics of gelatin gels.^48–50^ This trend remains consistent even with the incorporation of PEGDA, indicating that the addition of PEGDA does not alter the inherent temperature-dependent behavior of gelatin. An analogous behavior was observed for the other samples.

Figure 4 shows the gel time, obtained from the crossover of the viscoelastic moduli, as a function of the target temperature for all of the gelatin-based samples. The experimental data can be fitted with the Ross–Murphy equation:^44^

$$t_{g}=K_{T}{\left(1-\frac{T}{T_{c}}\right)}^{q}$$ 3

where *K*_*T*_ and *q* are fitting parameters. The curves represent the best fit through the data by using eq 3. The critical melting temperature, *T*_*c*_, is defined as the maximum temperature allowed for the gel before melting.^51^ Its value is close to the *T*_gel–sol_ measured in DTRTs at a rate of 1 °C/min (see Figure S7 in the Supporting Information), as this value approximates the ideal limit of zero heating rate.^47^ Table 2 presents the values of *T*_*c*_, *K*_*T*_, and *q* determined for each sample. Both parameters exhibit a strong dependence on the gelatin concentration. While the physical interpretation of *q* remains unclear, it is known that such a parameter is highly sensitive to *T*_*c*_.^51^ Conversely, *K*_*T*_ is expressed in time units and can be considered as the reciprocal of a reaction rate.

**Table 2:** Regression Parameters of eq 3 for the Experimental Data Shown in Figure 4

sample	*T*_*c*_ [°C]	*K*_*T*_ [s]	*q*
G2	26.0	61.1	–2.2
G6	31.0	7.4	–2.9
G2-P1	23.8	206	–2.25
G6-P1	28.8	155	–1.55
G6-P5	29.9	204	–1.36
G6-P10	28.4	193	–1.52

**Figure 4:**
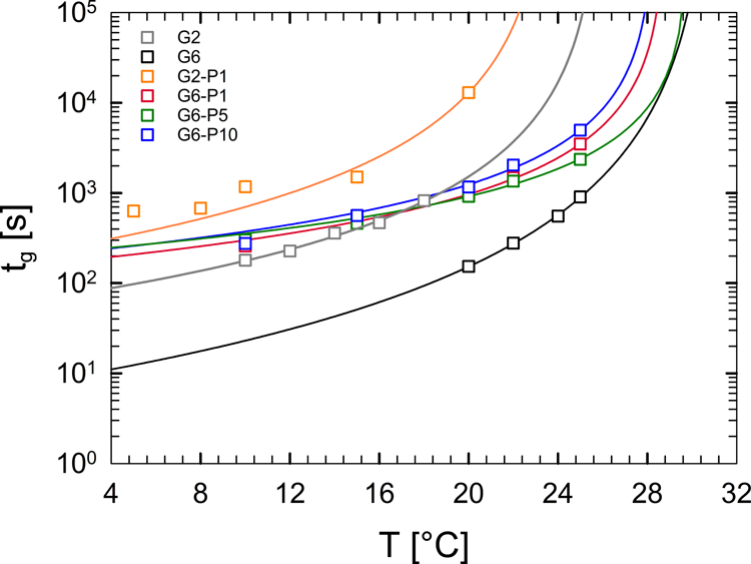
Ross–Murphy plot for gelatin and gelatin/PEGDA samples. Open symbols are the experimental and lines are the best fits obtained via eq 3.

As observed in the plot, all data closely follow the trend described by eq 3. The black symbols represent data sets for the pure gelatin/water solution at the same water-to-gelatin weight ratio as the hybrid solutions. Although the data of the pure gelatin aqueous solution and those of the corresponding hybrid solutions are well-described by eq 3, the addition of PEGDA has a significant effect on the gelation time, in that it increases the gelation time by 1 order of magnitude. As for nonisothermal gelation, the increase of the gelation time can be attributed to the ability of PEGDA to link both water molecules and gelatin. This hinders gelation by limiting the swelling of gelatin molecules and their association with triple helices. The data of Figure 4 also suggest that the variation of the PEGDA concentration between 1 and 10 wt % has a minimal effect in tuning the gelation time. This is likely due to the fact that the concentration of PEGDA is moderate. At higher concentrations (e.g., 25 wt %), PEGDA completely inhibits the gelation of gelatin (see Figure S5 in the Supporting Information).

### 3.3 Interplay of Physical and Chemical Gelation

To investigate the interplay between physical and chemical gelation, the isothermal gelation protocol described in Section 3.1 was employed, with the addition of UV irradiation. Specifically, the sample was cooled to a target temperature *T*_1_ and subsequently exposed to UV light through the glass plates of the rheometer while maintaining the temperature constant. Figure 5 presents the time evolution of the viscoelastic moduli of the three samples with a gelatin-to-water weight ratio equal to 6/94, and varying PEGDA concentrations, during isothermal gelation with and without UV curing at different target temperatures.

**Figure 5:**
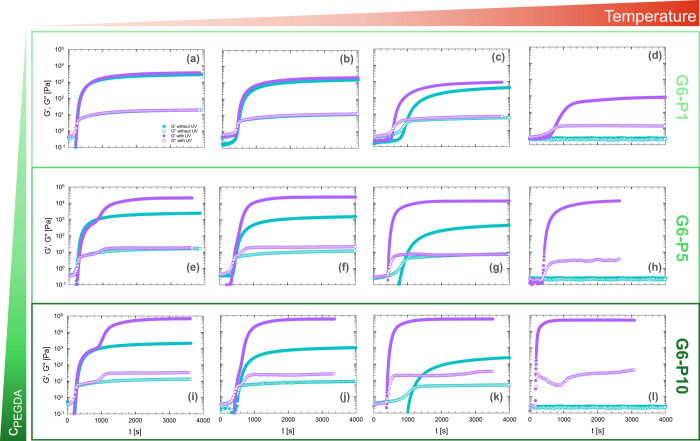
Comparison of the time evolution of *G*′ and *G*″ with (violet symbols) or without (teal symbols) UV curing, varying the concentration of PEGDA (G6-P1, G6-P5, and G6-P10) and temperature: (a, e, i) 10 °C; (b, f, j) 15 °C; (c, g, k) 20 °C, and (d, h, l) 60 °C. UV exposure was maintained for the entire duration of the test.

In the absence of UV light, the sol–gel transition is dominated by the physical gelation of gelatin, akin to the process described in Section 3.1 (teal symbols). When UV light is activated, chemical gelation occurs simultaneously with physical gelation (violet symbols).

Focusing, for example, on the sample G6-P1 in Figure 5, we observe that, at low temperatures (10 and 15 °C), the viscoelastic data of the experiments with and without UV irradiation virtually overlap, indicating that the physical gelation of gelatin dominates over chemical photo-cross-linking. The latter is relatively slow due to the low concentration of PEGDA (Figure 5a,b). However, as the target temperature is increased with respect to *T*_sol–gel_ of gelatin, physical gelation slows down, and the photo-cross-linking reaction becomes faster than the physical process. When the temperature is raised to 60 °C, physical gelation is completely suppressed; therefore, the elasticity of the resulting gel is provided exclusively by the chemically cross-linked network formed by the 1 wt % PEGDA present in the solution. Additionally, at this elevated temperature, gelatin has a lower viscosity, which facilitates the more efficient diffusion of free radicals within the system, further accelerating the polymerization process.

The behavior differs significantly at higher PEGDA concentrations, where the kinetics of physical and chemical gelation become comparable also at low target temperatures. At 10 °C, in the case of G6-P5 (Figure 5e), the two gelation mechanisms can be clearly distinguished. Initially, physical gelation dominates, as indicated by a shoulder in the curve corresponding to the characteristic plateau of the physically cross-linked hydrogel. This suggests that, at lower temperatures, gelatin’s ability to form a percolating network through hydrogen bonding and triple-helix formation is still effective, even in the presence of PEGDA. However, as the reaction progresses, chemical gelation gradually takes over, leading to a significantly stronger network. This transition is marked by the onset of an elastic plateau that is approximately 1 order of magnitude higher than that of the physical hydrogel, highlighting the superior mechanical properties imparted by covalent cross-linking.

As the temperature increases, the physical gelation process slows down, while chemical cross-linking becomes dominant. This difference becomes particularly evident as the chemical gel reaches a much higher plateau, nearly 2 orders of magnitude greater at 20 °C, suggesting that the physical interactions between gelatin chains contribute less to the overall mechanical properties under these conditions. This trend continues until *T*_*c*_ is reached. At 60 °C, as previously observed, the measured elastic plateau corresponds to that of the purely chemically cross-linked gel, confirming that the final mechanical properties at this temperature are dictated solely by the PEGDA network.

A similar trend is observed for sample G6-P10 (Figure 5i). In this case, the initial shoulder corresponding to the physical gel is more apparent, but the final modulus, controlled by the chemical cross-linking, is nearly 2 orders of magnitude higher than that of the physical gel. At 60 °C (Figure 5l), chemical gelation becomes the only relevant process.

### 3.4 Thermoreversibility of Hybrid Gels

Figure 6a,b shows the time evolutions of *G*′ and *G*″ for the same G6-P1 sample undergoing an identical thermal history. The only difference between the two experiments is the application of UV irradiation during the DTST, which is only presented in Figure 6b. As previously discussed and shown in Figure 5a, the gelation kinetics are identical in both cases.

**Figure 6:**
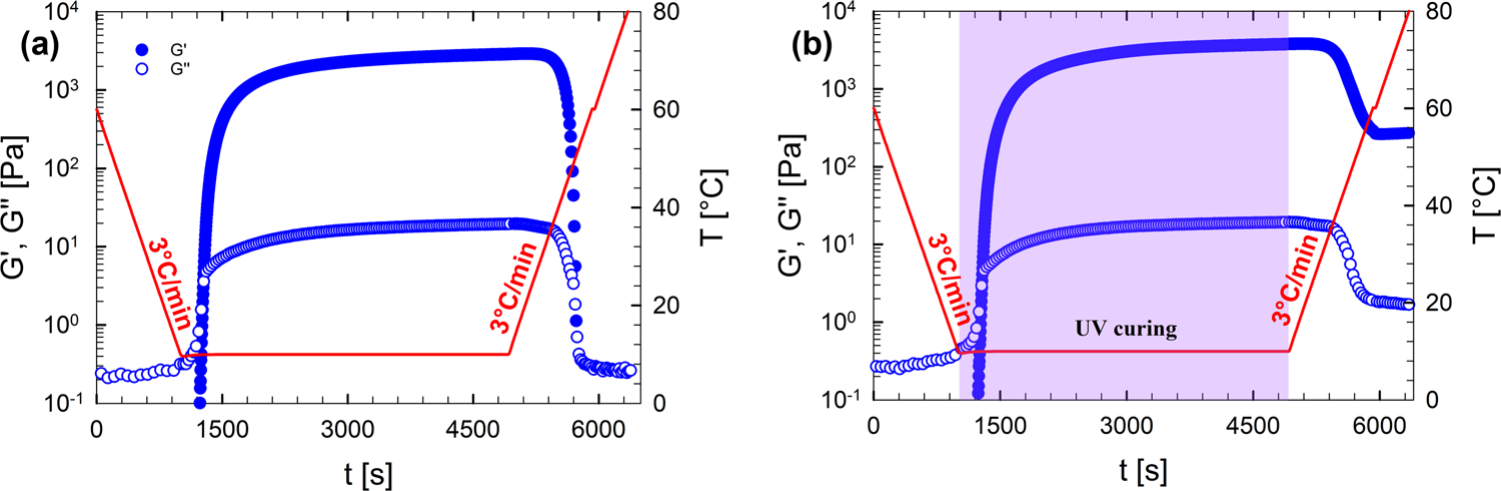
Time evolution of *G*′ and *G*″ for G6-P1 undergoing the thermal history indicated by the solid red line: cooling from 60 to 10 °C at 3 °C/min, a DTST at 10 °C performed either without (a) or with (b) UV curing, followed by heating to 80 °C at the same rate.

Significant differences emerge during the heating phase. In the case of exclusive physical gelation (Figure 6a), both moduli decrease, exhibit a crossover, and return to their initial values. This confirms the thermoreversible nature of the gelatin hydrogel. This behavior is consistent with the results reported in Figure 2b. In contrast, Figure 6b, featuring the viscoelastic properties of the sample cured with UV, shows that *G*′ and *G*″ initially decrease upon heating, due to the disruption of gelatin’s triple helices into random coil conformations. However, the moduli do not return to their initial values but reach a final value that matches those observed at 60 °C for the fully chemically cross-linked hydrogel, thus confirming the different roles of the two networks.

In this work, a relatively low UV irradiance of 0.4 mW/cm^2^ was employed to ensure homogeneous curing while avoiding thermal effects and potential photodegradation. It is known that higher irradiance can enhance the rate and extent of polymerization,^52,53^ reducing the required exposure time. However, as reported by Zhang et al.,^54^ increasing the UV intensity beyond a certain threshold (e.g., 5 mW/cm^2^) may lead to degradation of PEGDA hydrogels, inhomogeneous cross-linking across the sample thickness, and undesirable optical and mechanical effects. Therefore, the selected intensity represents a compromise between the curing efficiency and network quality.

### 3.5 Effect of Gelatin and PEGDA Concentration

Figure 7 presents |G*| of the hybrid solutions as a function of *t*/*t*_*g*_ under UV curing at specific target temperatures.

**Figure 7:**
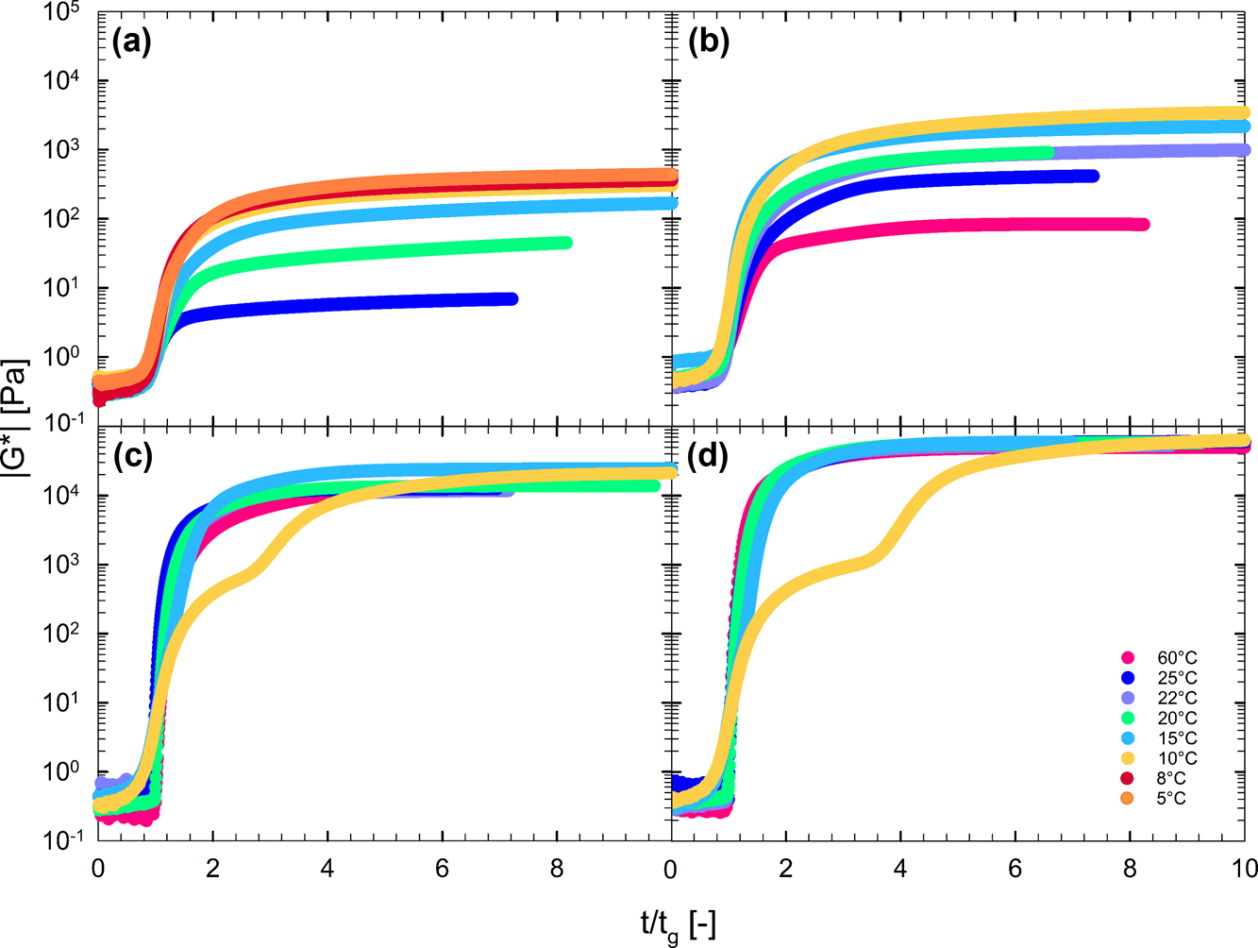
Complex modulus, |G*|, as a function of *t*/*t*_*g*_ under UV curing for different samples: (a) G2-P1, (b) G6-P1, (c) G6-P5, and (d) G6-P10. UV curing was maintained for the entire duration of the test.

For the two samples with 1 wt % of PEGDA (G2-P1, G6-P1), the final elastic plateau value at long times depends on the temperature at which the DTST is performed. This behavior indicates that the formation of the cross-linked network is due to gelatin only, as expected given the low PEGDA concentration.

It is worth noticing that for G2-P1, photopolymerization does not occur at 60 °C, in contrast to what happens for G6-P1, suggesting that, at low PEGDA concentrations, the presence of gelatin is essential for enabling network formation even in the case of pure chemical gelation. This is further corroborated by the observation that a formulation containing only 1 wt % PEGDA and 1 wt % of I2959 in water fails to photopolymerize under the same conditions (see Figure S1 in the Supporting Information). Such a synergistic effect can be attributed to the ability of gelatin to stabilize free radicals through its carboxyl and hydroxyl functional groups. Given the low concentration of PEGDA, this stabilization plays a crucial role in preventing rapid radical deactivation and promoting effective gelation. The curve at 60 °C is absent because photopolymerization does not occur within the experimental time frame.

In contrast to the behavior observed at lower PEGDA concentrations, for G6-P5 and G6-P10, the elastic plateau value does not depend on the target temperature. This indicates that, at such concentrations of PEGDA, the chemical cross-linking process is dominant. The effect of the interplay of physical and chemical gelation on the elasticity of the resulting gels is summarized in Figure 8a, where the plateau modulus of the gels as a function of target temperature for the different samples is reported. At low target temperatures and PEGDA concentration equal to 1 wt %, the elasticity is dominated by the physical network of gelatin. As a result, the modulus is relatively low and depends on the target temperature. At high PEGDA concentrations, the elasticity is governed by the chemical network of PEGDA. The elastic modulus is higher and nearly independent of the target temperature as the kinetics of the photopolymerization process are nearly temperature-independent.

**Figure 8:**
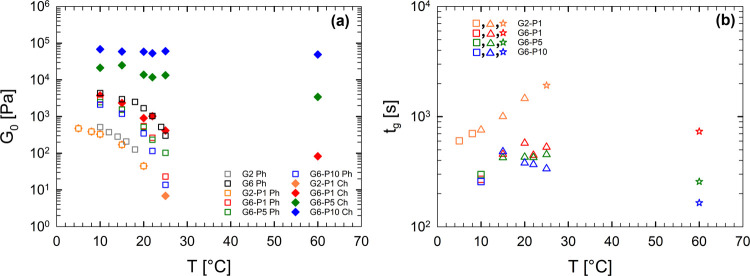
(a) Plateau value of the elastic modulus, *G*_0_, as a function of the temperature. The legend distinguishes between physical (Ph) and chemical (Ch) gelation mechanisms. (b) Gelation time determined from DTSTs under UV curing as a function of temperature. Each symbol represents a distinct gelation mechanism: squares correspond to purely physical gelation, triangles indicate concurrent physical and chemical gelation occurring in parallel, and stars denote purely chemical gelation.

Figure 8b presents the gelation time as a function of temperature, with distinct symbols indicating the different gelation mechanisms. Squares correspond to conditions where physical gelation due to gelatin dominates, and the two kinetic processes occur sequentially. Triangles represent scenarios in which physical and chemical gelation mechanisms act in parallel and enhance each other. Stars indicate purely chemical gelation. By playing with target temperature, composition, and gelation kinetics, apart from different elastic properties, a large variety of gelation times can be achieved.

### 3.6 Morphology of the Hydrogels

Figure 9 displays SEM images of the freeze-dried hydrogels. The top row (a–d) shows images of the pure gelatin/water and PEGDA/water hydrogels, namely, G2, G6, P5, and P10.

**Figure 9:**
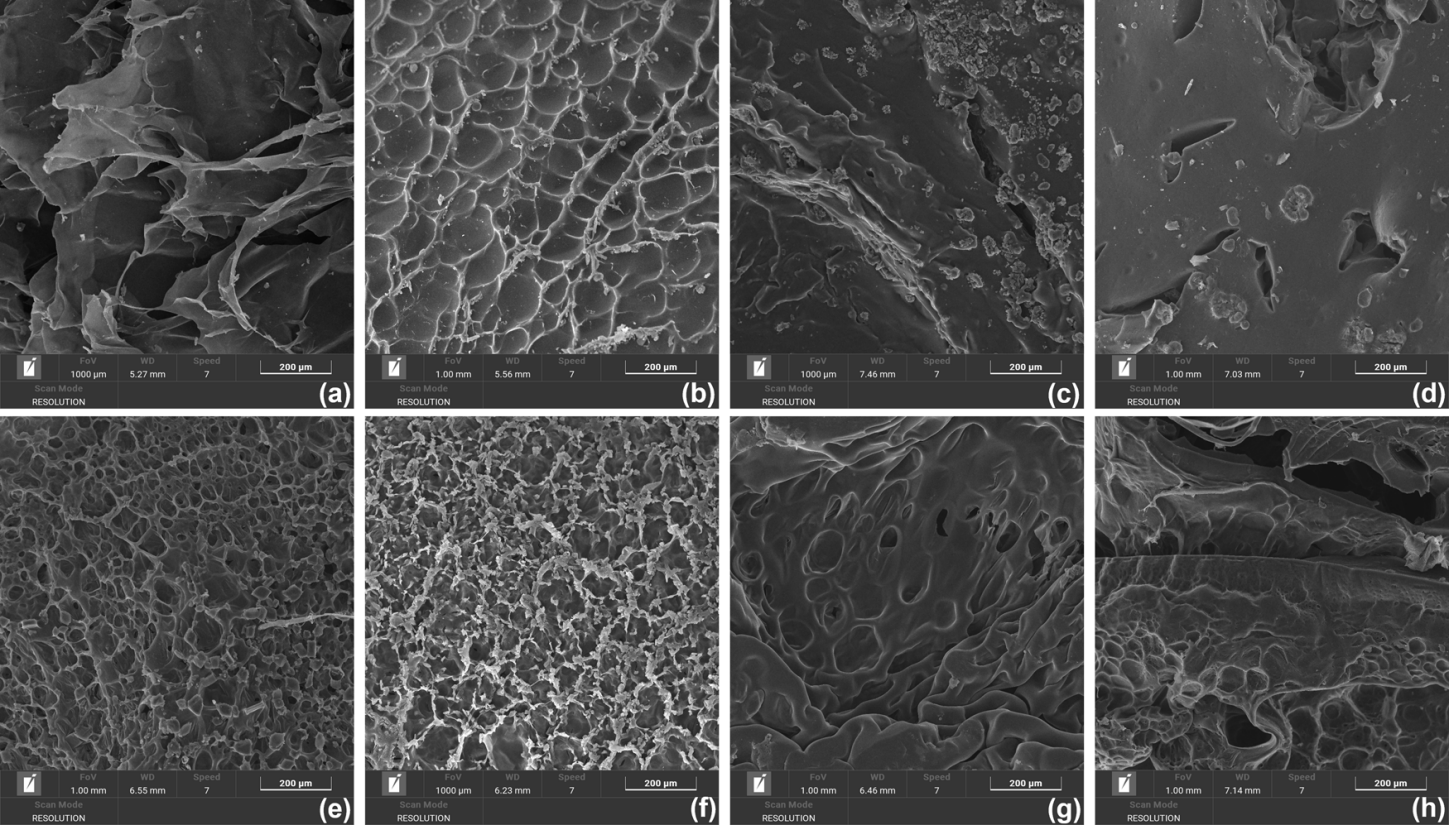
SEM images of (a) G2, (b) G6, (c) P5, (d) P10, (e) G2-P1, (f) G6-P1, (g) G6-P5, and (h) G6-P10 (scale bar: 200 μm).

In the case of the gelatin gels, G2 exhibits a highly porous and flaky morphology, whereas G6 shows a more compact structure with smaller, more closed pores. In contrast, the pure PEGDA hydrogels, P5 and P10, display a denser and smoother architecture.

The bottom row (e–h) displays four hybrid hydrogels that were photopolymerized under the rheometer, following the thermal protocol described in the Methods section with *T*_1_ = 10 °C.

All samples exhibit a porous microstructure, with clear trends related to the PEGDA and gelatin content. Hydrogels with lower PEGDA concentration show a more porous architecture, characterized by numerous small pores, whereas increasing the PEGDA content results in a denser and less porous structure, progressively resembling the morphology of the pure PEGDA hydrogels P5 and P10 (Figure 9c,d). Such morphological features are consistent with the viscoelastic moduli observed in the hybrid gels. Samples with higher PEGDA content, such as G6-P5 and G6-P10, result in more elastic networks with reduced porosity.

To investigate the influence of polymerization temperature on the microstructure, we analyzed the same hybrid hydrogel at low PEGDA content (G6-P1), known to exhibit temperature-dependent behavior, after polymerization at different temperatures. As shown in Figure 10, increasing the temperature leads to more compact morphologies, despite a decrease in elastic modulus. This apparent contradiction with the previous findings can be explained by considering that a significant fraction of pores could have sizes small enough to be not resolvable by imaging techniques (as also pointed out in Section 3.8 for other samples). Most probably, photopolymerizing at higher temperatures reduces the extent of large pores but increases that of small ones, potentially correlating with a lower elastic modulus.

**Figure 10:**
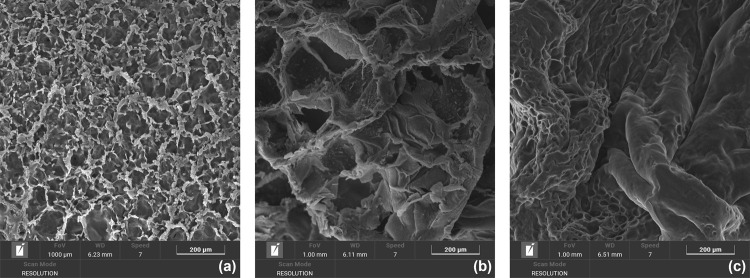
SEM images of G6-P1 photopolymerized at different temperatures: (a) 10 °C, (b) 20 °C, and (c) 60 °C (scale bar: 200 μm).

These findings highlight the critical interplay between physical and chemical cross-linking in determining hydrogel morphology and mechanical behavior and underscore the potential of polymerization temperature as a design parameter for tuning hybrid hydrogel properties.

### 3.7 Swelling Ratio and Gel Fraction

The SEM and rheological measurements reported above indicate that a denser gel network is formed as the PEGDA concentration increases in the prepolymerized solution. As a consequence, systems with a lower PEGDA content are expected to show an enhanced capacity to absorb water.

The influence of gelatin and PEGDA composition on the swelling ratio of hybrid hydrogels in bidistilled water at 25 °C is shown in Figure 11. Notably, the PEGDA-only hydrogel with 1 wt % PEGDA is absent, as previously mentioned, due to its inability to undergo photopolymerization. Consequently, the comparison is limited to hydrogels with higher PEGDA concentrations.

**Figure 11:**
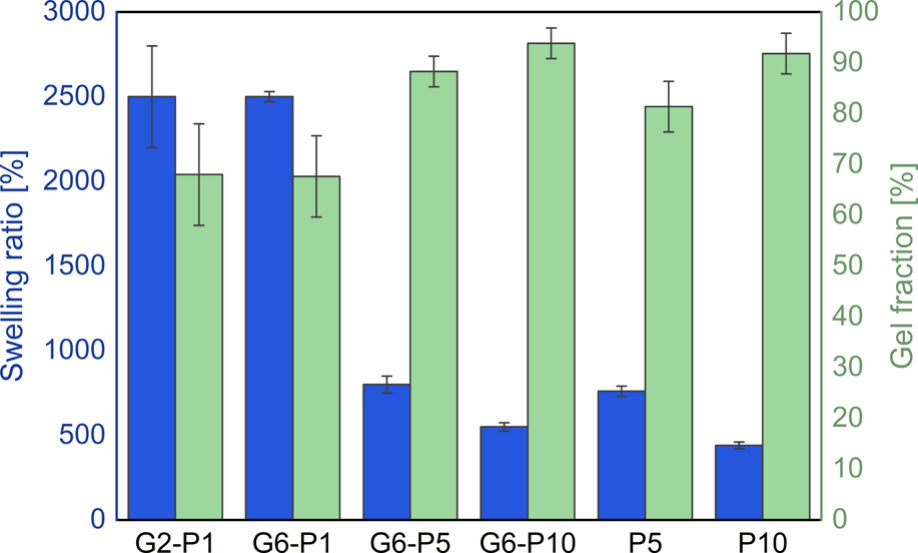
Equilibrium swelling ratio after immersing in bidistilled water for 48 h, left *y*-axis, and gel fraction, right *y*-axis, as functions of the composition of gelatin and PEGDA at 25 °C. Error bars are shown along with data for each sample, representing the standard error of multiple measurements.

As expected, the equilibrium swelling ratio increases with a decreasing concentration of PEGDA. Moreover, the results indicate that the swelling of G6-P5 and P5 is similar, as is the case for G6-P10 and P10. This trend aligns with previous observations (Figure 7c,d), which demonstrated that the chemically cross-linked network dominates over physical interactions, making PEGDA the primary structural component.

The two samples with 1 wt % of PEGDA exhibited equivalent swelling ratios, which is not unexpected given their comparable elastic plateau modulus and morphological structure. The elevated error bar for G2-P1 is attributable to the hydrogel’s increased fragility and the associated challenges in precise weighing.

These swelling results are also consistent with the determination of the gel fraction via eq 2, which is a qualitative measure of network formation efficiency, reflecting the extent of covalent cross-linking between polymer chains.^55^ As the PEGDA content increases, a greater number of covalent cross-links are formed within the hydrogel network, resulting in a higher gel fraction. A more cross-linked network restricts the mobility of polymer chains and reduces the hydrogel’s ability to absorb water, thereby explaining the lower swelling ratios and higher gel fractions observed in samples with higher PEGDA concentrations. Moreover, consistent with previous studies,^36^ hybrid hydrogels containing gelatin exhibited a higher gel fraction compared to those without gelatin in photopolymerized systems, highlighting the beneficial role of gelatin in enhancing network integrity.

### 3.8 Skeletal Density

Figure 12 presents the skeletal densities of all freeze-dried hydrogels, as determined by helium pycnometry. For pure PEGDA hydrogels, the densities are clearly higher than that of pure water. Although based on only two formulations, the data suggest that the density increases with higher PEGDA concentrations in the initial solution.

**Figure 12:**
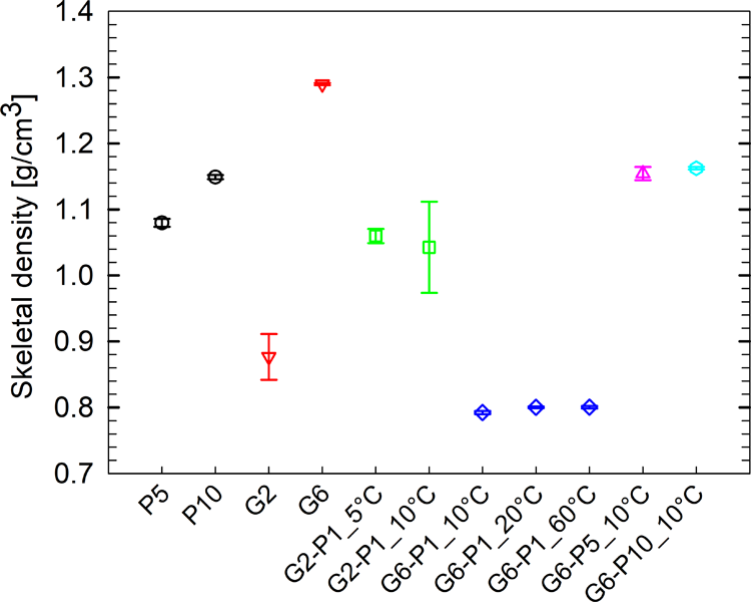
Skeletal densities determined via helium pycnometry for all hydrogels formulations. The temperatures indicated next to each sample name refer to the photopolymerization temperature of the corresponding hydrogel.

A similar trend is observed in gelatin-based samples, although the differences in density are more pronounced compared with pure PEGDA-based ones. Specifically, the sample with a lower gelatin concentration exhibits a density below that of pure water, while the sample with a higher gelatin concentration has a density exceeding that of pure PEGDA-based samples. This result is consistent with the findings reported by Van Vlierberghe et al.,^56^ who pointed out a remarkable porosity decrease (i.e., density increase) at higher gelatin concentrations due to the lack of pores that are easily permeated by helium but hardly, if not at all, resolved by imaging investigations (SEM, microcomputed tomography).

Interestingly, this trend reverses in hybrid samples with a low PEGDA content. Indeed, the densities of G2-P1 samples are significantly higher than those of the G6-P1 samples. A similar pattern can be inferred from the results of Fu et al.,^57^ who investigated the drug release kinetics of semi-interpenetrating networks (sIPNs) of gelatin and PEGDA. The study examined sIPNs with varying gelatin/PEGDA weight ratios. It was observed that increasing gelatin content led to higher initial solute release rates and intragel diffusivity. This suggests that higher gelatin concentrations result in a more porous network, potentially correlating with a lower density. It is also notable that the density of the G6-P1 samples remains largely unaffected by the photopolymerization temperature. As already pointed out in Section 3.6, increasing the photopolymerization temperature presumably reduces the extent of large pores but increases that of small ones, potentially leading to a compensation in terms of porosity and thus density.

Further examination of Figure 12 reveals that increasing the PEGDA content up to concentrations that are like or even higher than that of gelatin leads again to higher density values. Indeed, Testore et al. already found that, in photopolymerized hybrid hydrogels, higher PEGDA/gelatin ratios yield more cross-linking points per unit volume.^35^ This indicates a tighter network and, thus, a higher polymer density. Finally, it is worth noting that once the gelatin/PEGDA weight ratio approaches unity, further increases in PEGDA concentration do not seem to cause significant corresponding changes in sample density.

## 4 Conclusions

This study systematically investigated the interplay between physical and chemical gelation in gelatin/PEGDA hybrid hydrogels, providing key insights into how temperature, polymer concentration, and UV exposure control the kinetics and mechanical properties of the resulting gels.

The gelation temperature and hysteresis are strongly influenced by the gelatin concentration, with higher concentrations promoting more defined and robust physical networks. Isothermal tests confirm that physical gelation follows predictable kinetics well-described by the Ross–Murphy model, and that PEGDA, while not altering the temperature-dependent behavior of gelatin, significantly affects the gelation time and final network strength.

Upon UV activation, chemical gelation through PEGDA cross-linking enhances the hydrogel’s stiffness, especially at higher PEGDA concentrations, and dominates at elevated temperatures where physical gelation is suppressed. The transition from thermoreversible to thermoirreversible behavior is clearly demonstrated, and the mechanical fingerprint of the chemically cross-linked network is consistently reproducible.

SEM and skeletal density analyses reveal composition-dependent differences in microstructure and porosity, confirming that the hydrogel architecture is tunable through formulation and processing parameters.

Importantly, this dual responsiveness enables precise control over gelation kinetics, mechanical strength, and the tunability of hybrid hydrogel properties through a controlled variation of formulation parameters. Moreover, this sensitivity of gelation dynamics provides a valuable framework for optimizing hydrogel behavior in situ. This offers a versatile platform for the design of advanced biomaterials with tailored performance. From an application perspective, the ability to independently modulate stiffness, gelation time, and microstructure positions these hydrogels as highly promising for biomedical engineering, including injectable scaffolds, drug delivery systems, and tissue regeneration platforms.

## Supplementary Material



## Data Availability

The data that support the findings of this study are available from the corresponding author upon request.

## Notes

Additional rheological data, time-sweep tests on pure PEGDA hydrogels, frequency-dependent viscoelastic properties of selected formulations, temperature-dependent gelation behavior from cooling ramps and isothermal tests, and high-resolution SEM images (PDF)
