# Supplementary material for: Design and Characterization of Hybrid Gelatin/PEGDA Hydrogels with Tunable Viscoelastic Properties
Source: Biomacromolecules. 2025 Jul 25;26(8):5450–60. doi: 10.1021/acs.biomac.5c01048 (PMC12344689; doi:10.1021/acs.biomac.5c01048)
Supplement: Supplementary file 1 [file bm5c01048_si_001.pdf]

Design and characterization of hybrid gelatin/PEGDA hydrogels with tunable viscoelastic properties

Pietro Renato Avallone,[*,†,¶] Nadia Russo,[†,¶] Nicola Gargiulo,[†] Nino Grizzuti,[‡] and Salvatore Costanzo[‡]

†*Center of Advanced Measurement and Technology Services (CeSMA), Federico II University, Corso N. Protopisani, Naples, 80146, Italy.*

‡*Department of Chemical, Materials, and Industrial Production Engineering, Federico II University, P.le V. Tecchio 80, 80125, Naples, Italy*

¶*Contributed equally to this work*

E-mail: pietrorenato.avallone@unina.it

Supporting Information Available

PEGDA in water

Fig. S1 presents the evolution of the elastic modulus over time for three samples containing different concentrations of PEGDA in water during in situ photopolymerization at 25°C. The formulation with the lowest PEGDA content (1 wt%) does not appear to undergo photopolymerization within the investigated time frame. In contrast, higher PEGDA concentrations result in successful hydrogel formation, with increased polymer content accelerating the gelation process. Additionally, samples with higher PEGDA concentrations exhibit greater plateau values of the elastic modulus

at long times, indicating the formation of networks with higher chemical crosslinking density and improved elastic properties.

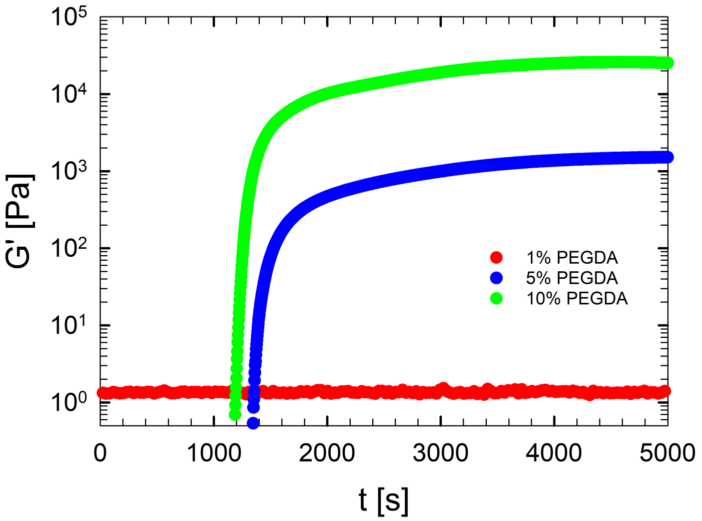

Figure S1: Storage modulus, G′, as function of time at 25°C for pure PEGDA hydrogels, parametric in PEGDA weight concentration.

Dynamic frequency sweep tests

Fig. S2 illustrates the complex viscosity as a function of angular frequency ω for a representative sample. The results indicate that the material exhibits Newtonian behavior at 60°C.

Fig. S3 and S4 displays the viscoelastic moduli as functions of ω at different temperatures for G2-P1 and G6-P5, respectively.

Dynamic temperature ramp test

Fig. S5 presents the cooling ramp of a sample containing 25 wt% of PEGDA. The plot reveals that this concentration of PEGDA suppresses the physical gelation of gelatin.

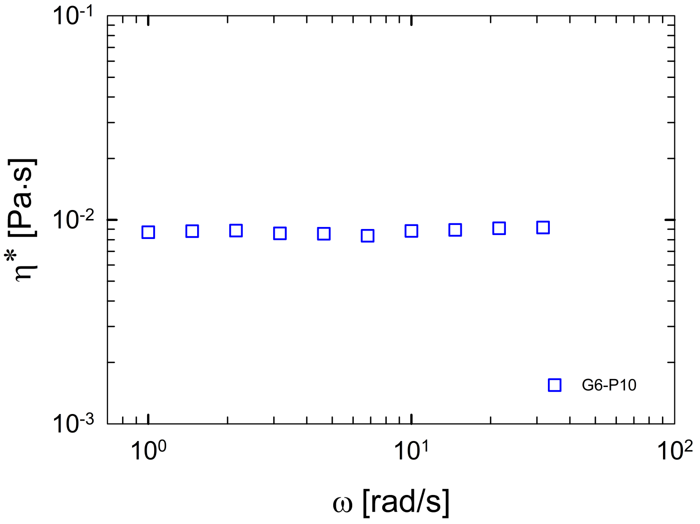

Figure S2: Complex viscosity, $\eta^\star$, as functions of angular frequency, ω, for G6-P10 sample at 60°C.

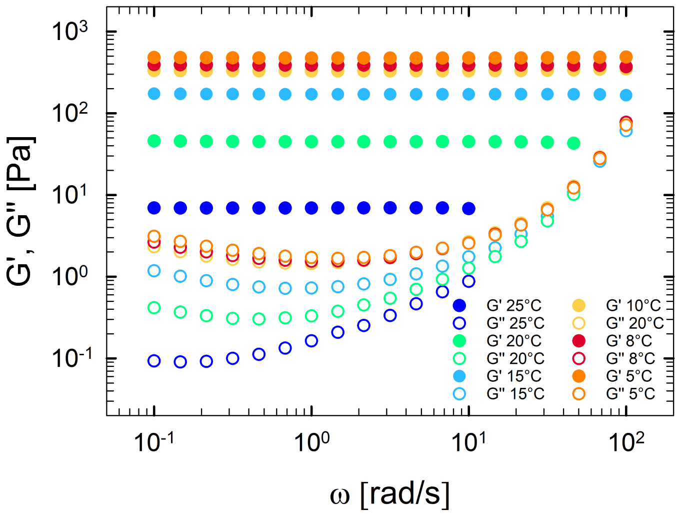

Figure S3: Storage modulus, G′, and loss modulus, G″ as functions of angular frequency, ω, at different temperatures for G2-P1.

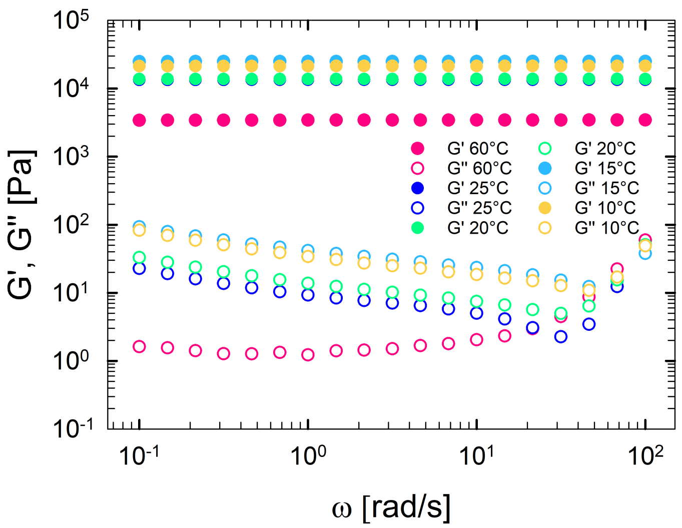

Figure S4: Storage modulus, G′, and loss modulus, G″ as functions of angular frequency, ω, at different temperatures for G6-P5.

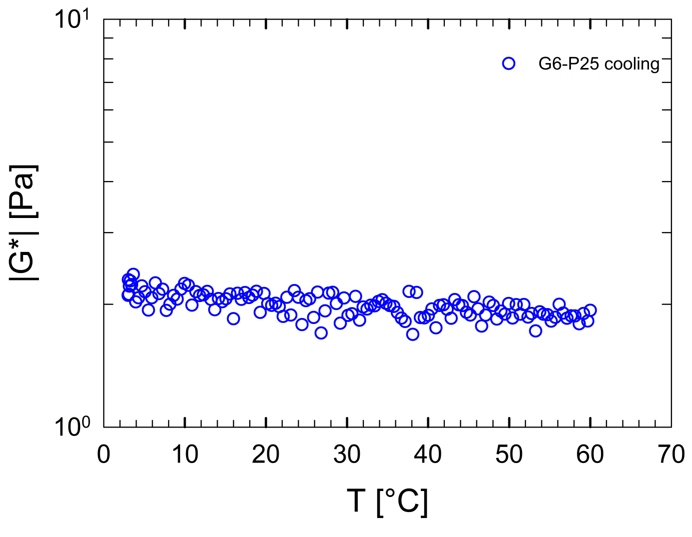

Figure S5: Temperature dependence of the complex modulus for the sample containing 25 wt% PEGDA (G6-P25), measured during a cooling ramp at 3°C/min.

Physical gelation under isothermal conditions

Each sample was loaded and thermally equilibrated at 60°C, then it was cooled down to a target temperature, T_1, at fixed cooling rate (3°C/min), and the viscoelastic moduli were measured as functions of time. The UV light was kept off for the entire duration of the experiment. As an example, Fig. S6 presents the time evolution of G′ and G″ for samples G2 and G6 at different target temperatures.

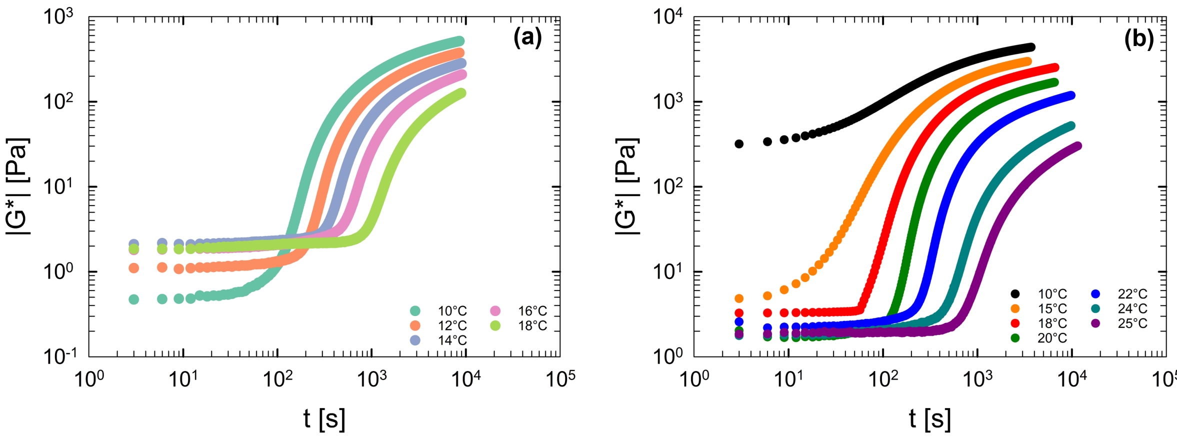

Figure S6: Evaluation of isothermal gelation kinetics for G2 and G6 sample at different temperatures.

Dynamic temperature ramp tests at 1°C/min

Fig. S7 displays the viscoelastic moduli of the hybrid samples as a function of temperature, measured at a constant heating rate of 1°C/min. The critical melting temperature T_c was determined from these measurements.

Dynamic time sweep test

Fig. S8 displays the time evolution of the complex modulus $|G^*|$ for the G2-P1 sample at different target temperatures. It is particularly evident that at 60°C, where gelatin does not undergo physical gelation, photopolymerization does not proceed.

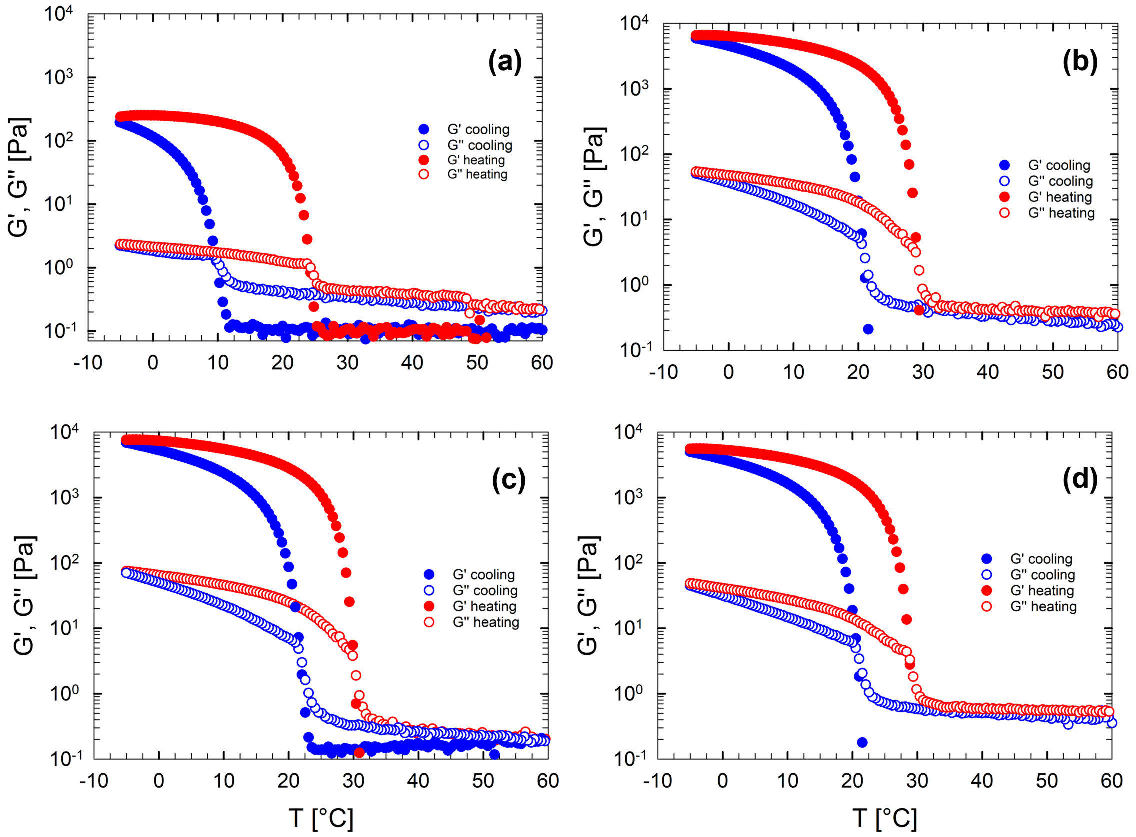

Figure S7: Storage modulus G′ and loss modulus G″ as functions of the temperature for the sample (a) G2-P1, (b) G6-P1, (c) G6-P5 and (d) G6-P10 at 1°C/min.

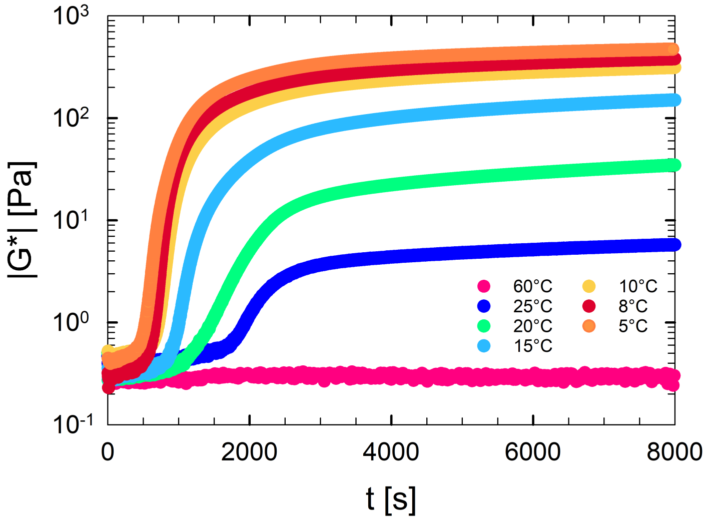

Figure S8: Complex modulus as function of the time for G2-P1 at different target temperatures.

High-resolution SEM images

The following figures (Figures S9 to S18) present the individual high-resolution images that compose Figures 9 and 10 in the main text.

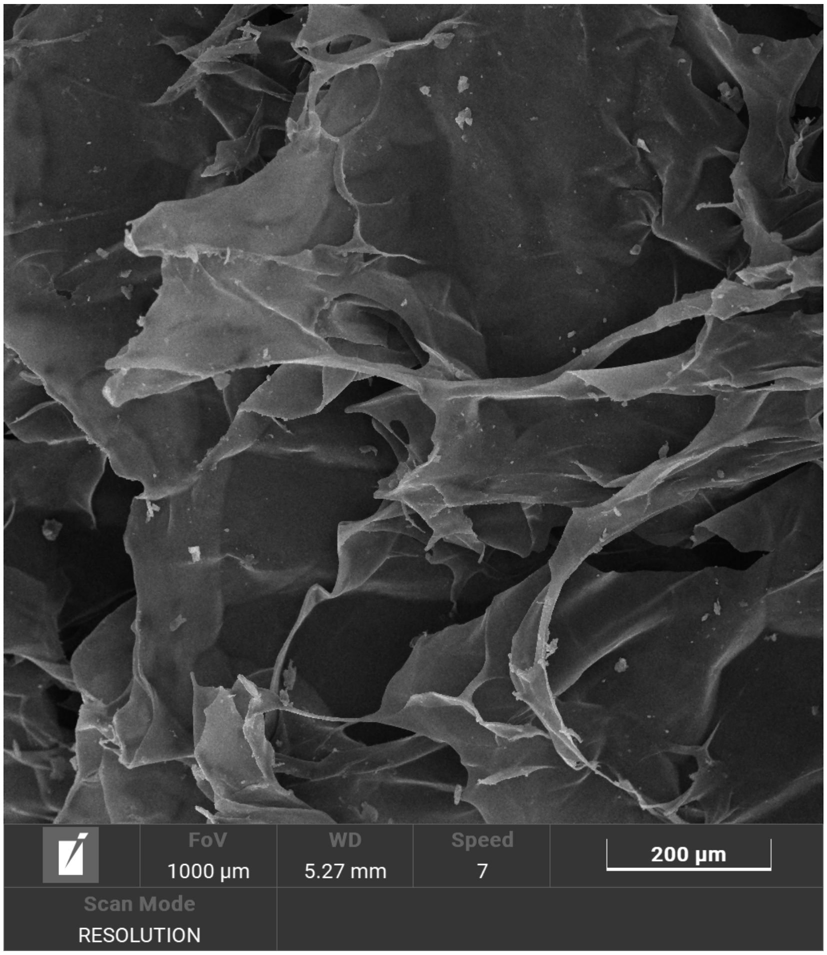

Figure S9: High-resolution SEM image of sample G2, shown in the main text as Figure 9a.

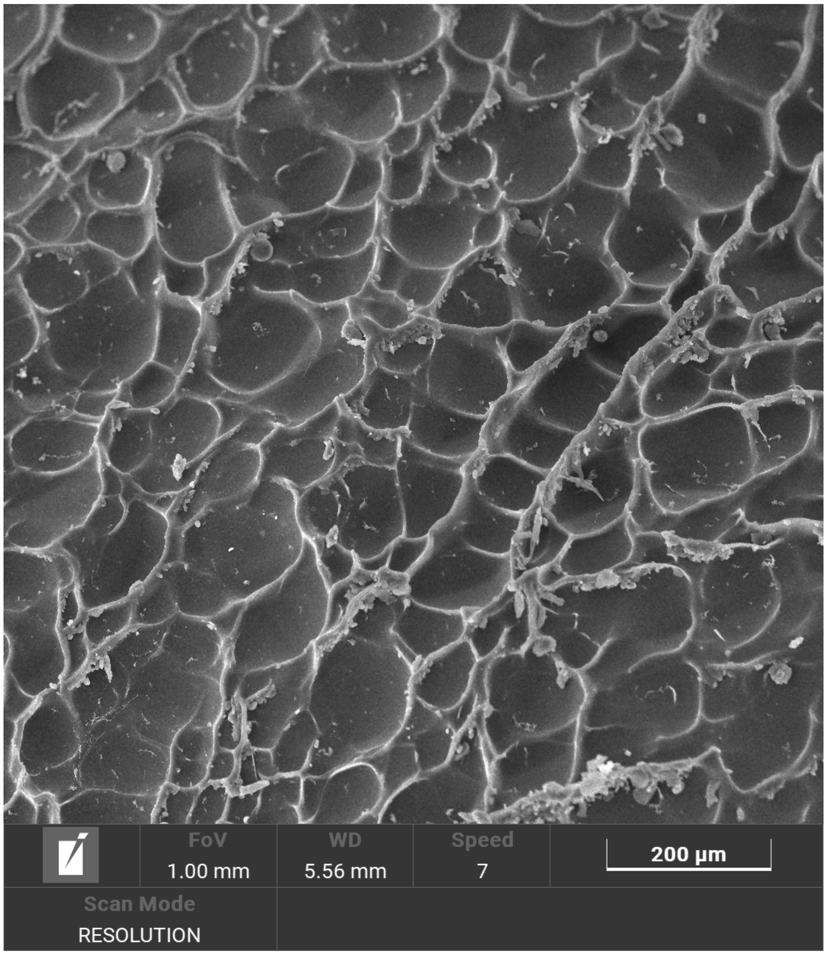

Figure S10: High-resolution SEM image of sample G6, shown in the main text as Figure 9b.

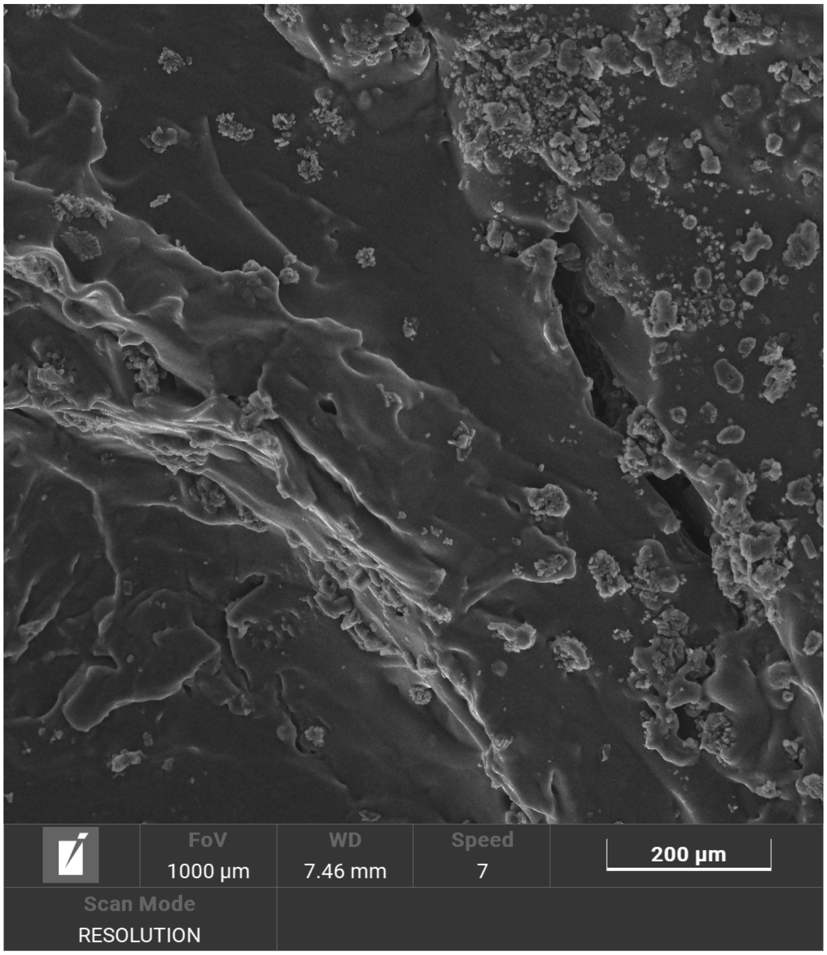

Figure S11: High-resolution SEM image of sample P5, shown in the main text as Figure 9c.

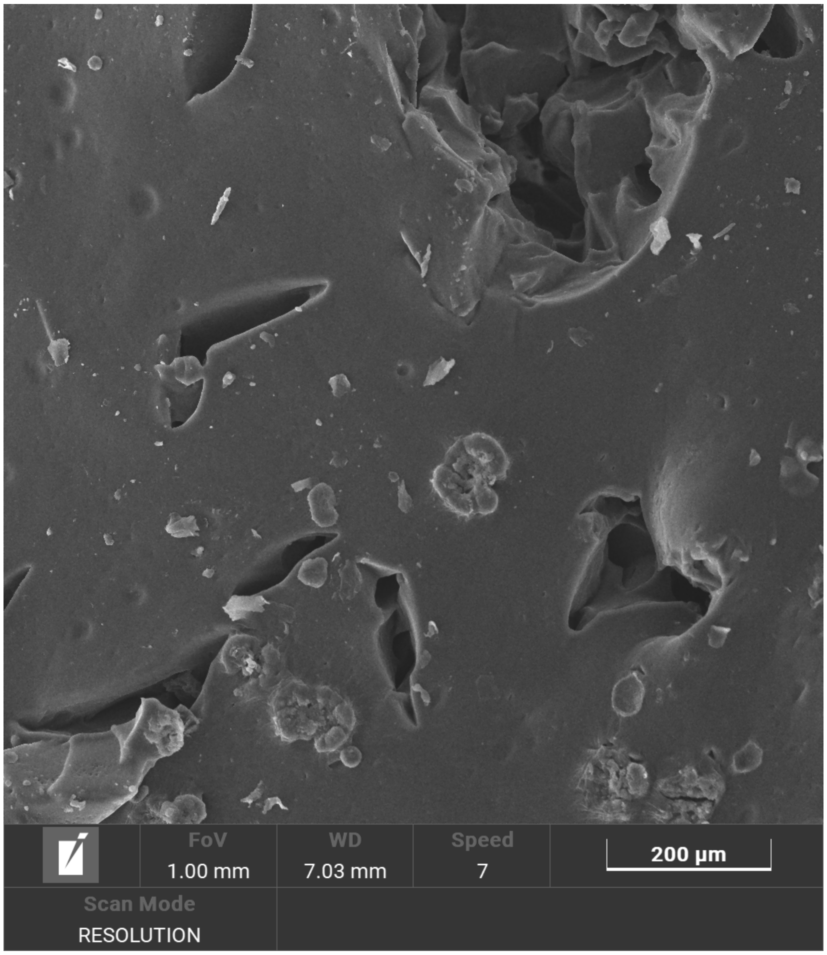

Figure S12: High-resolution SEM image of sample P10, shown in the main text as Figure 9d.

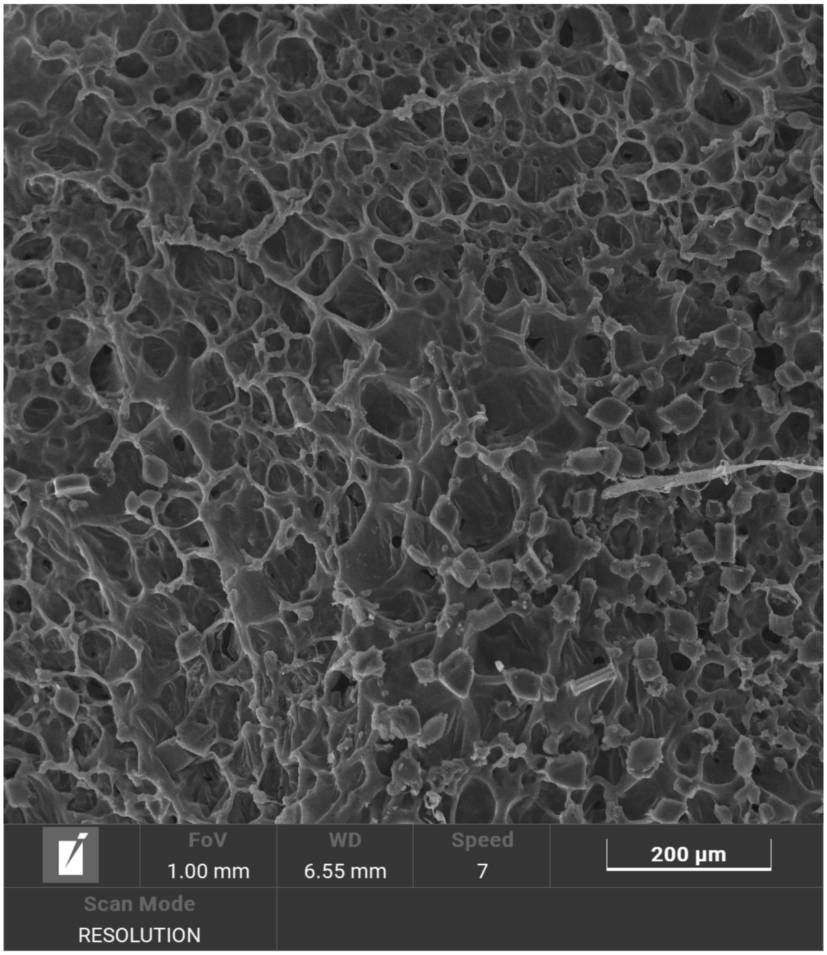

Figure S13: High-resolution SEM image of sample G2-P1, shown in the main text as Figure 9e.

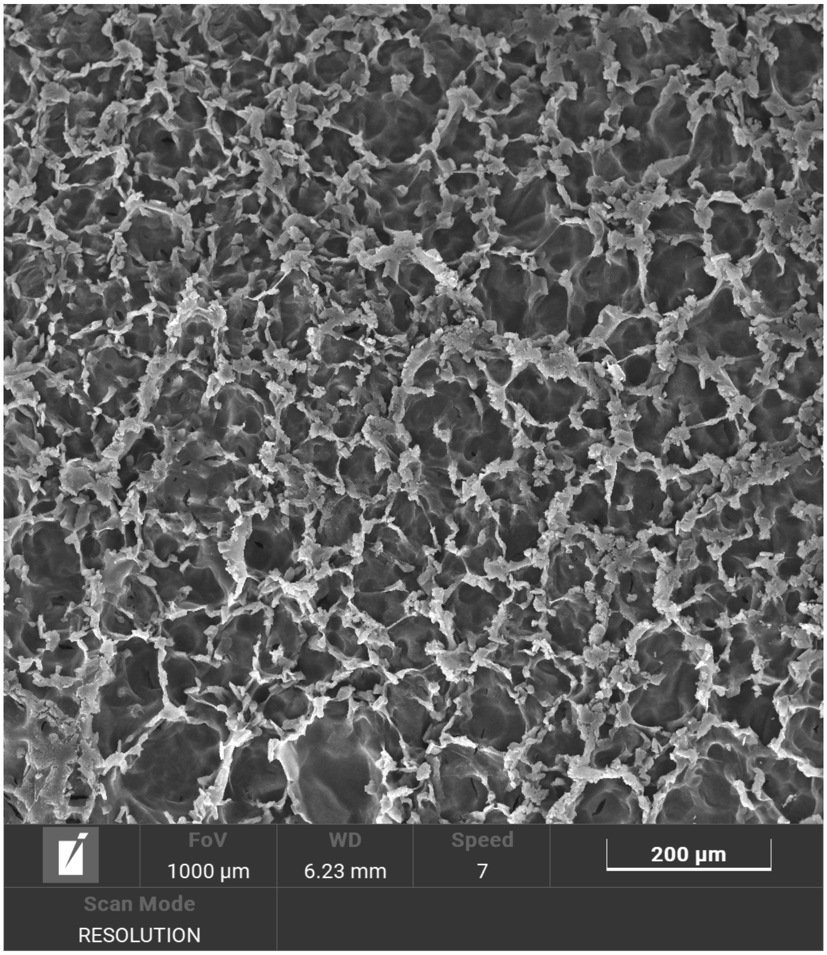

Figure S14: High-resolution SEM image of sample G6-P1, shown in the main text as Figure 9f and Figure 10a.

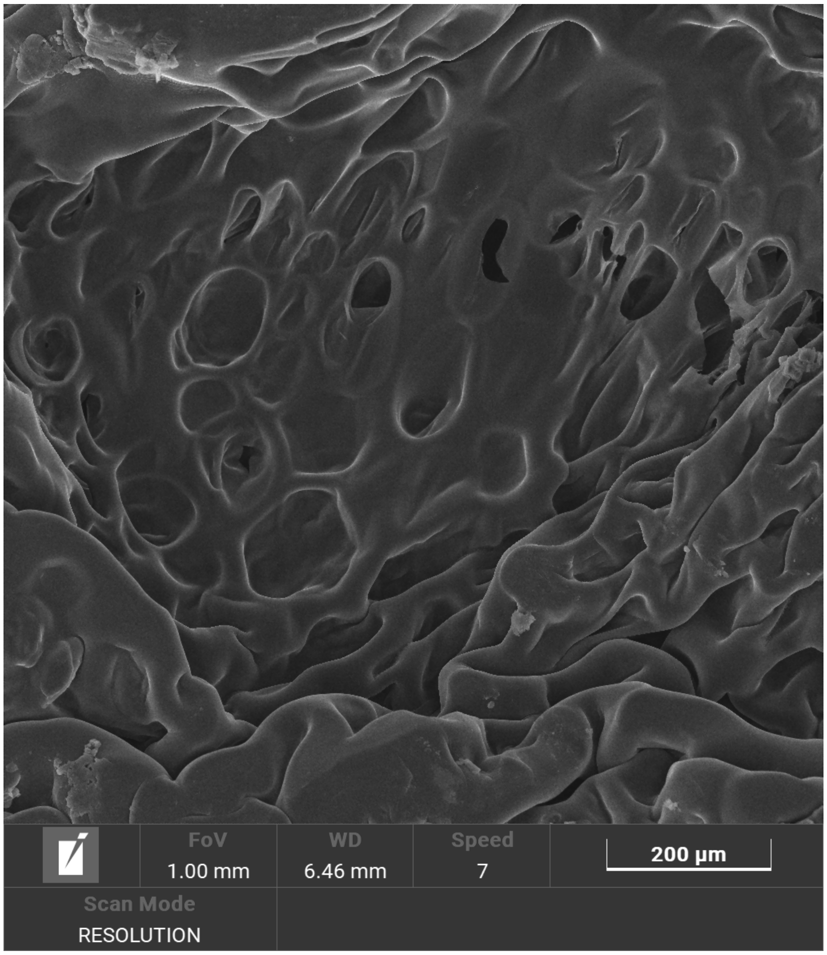

Figure S15: High-resolution SEM image of sample G6-P5, shown in the main text as Figure 9g.

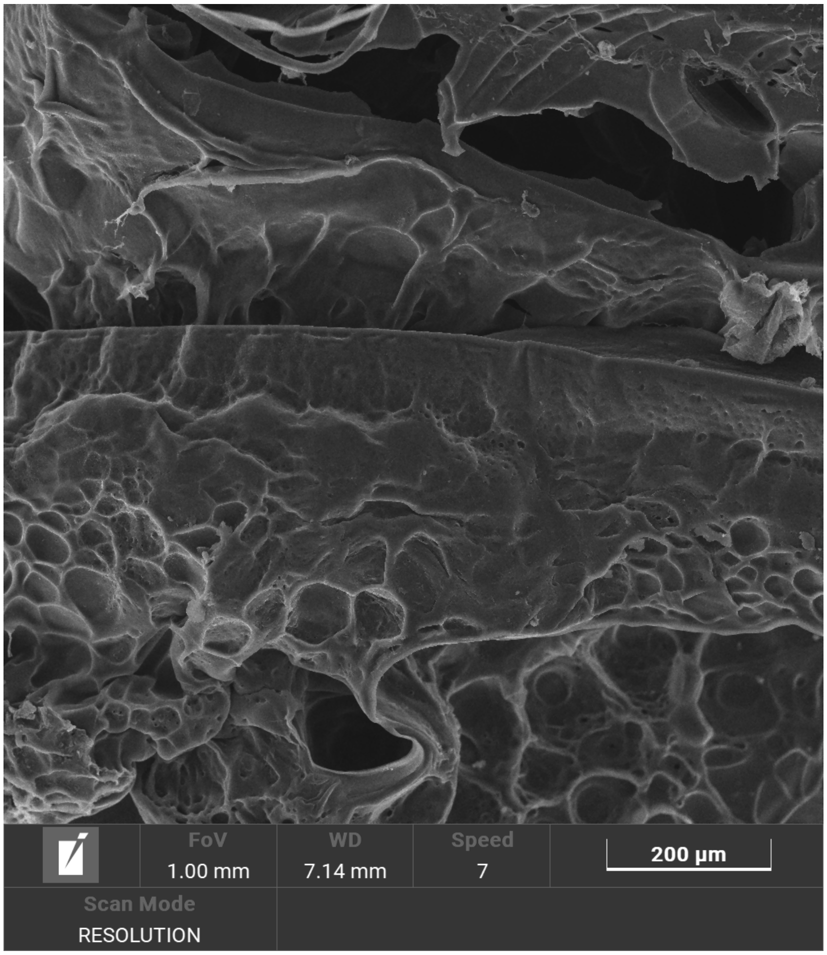

Figure S16: High-resolution SEM image of sample G6-P10, shown in the main text as Figure 9h.

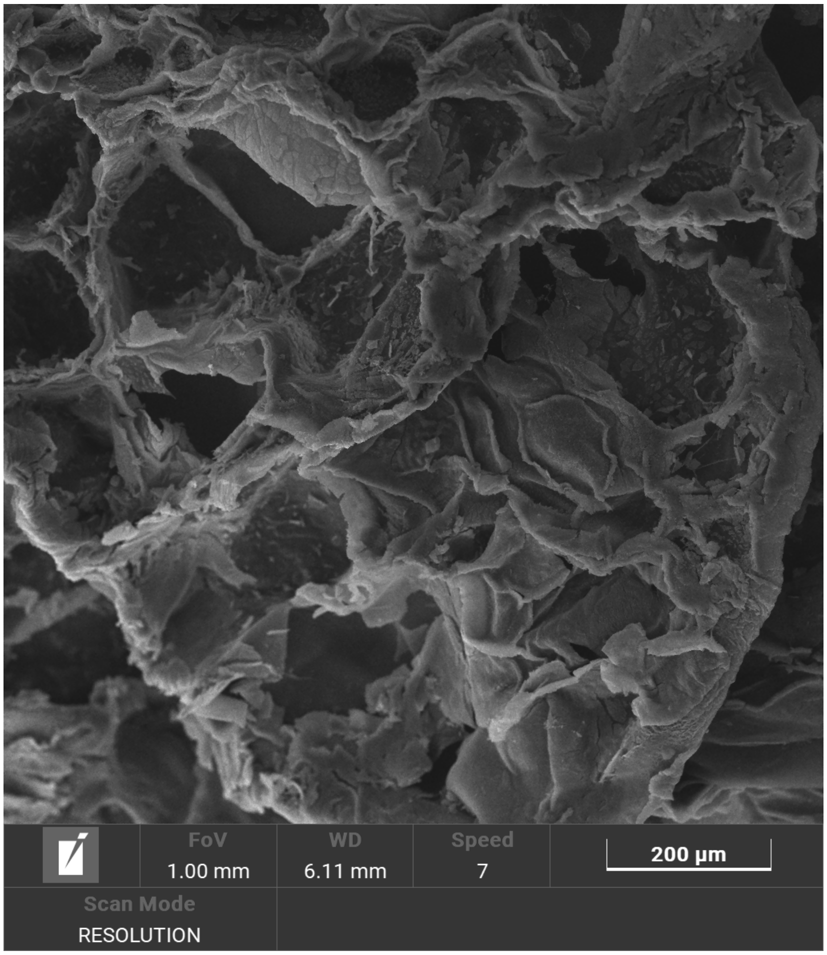

Figure S17: High-resolution SEM image of sample G6-P1 photopolymerized at 20°C, shown in the main text as Figure 10b.

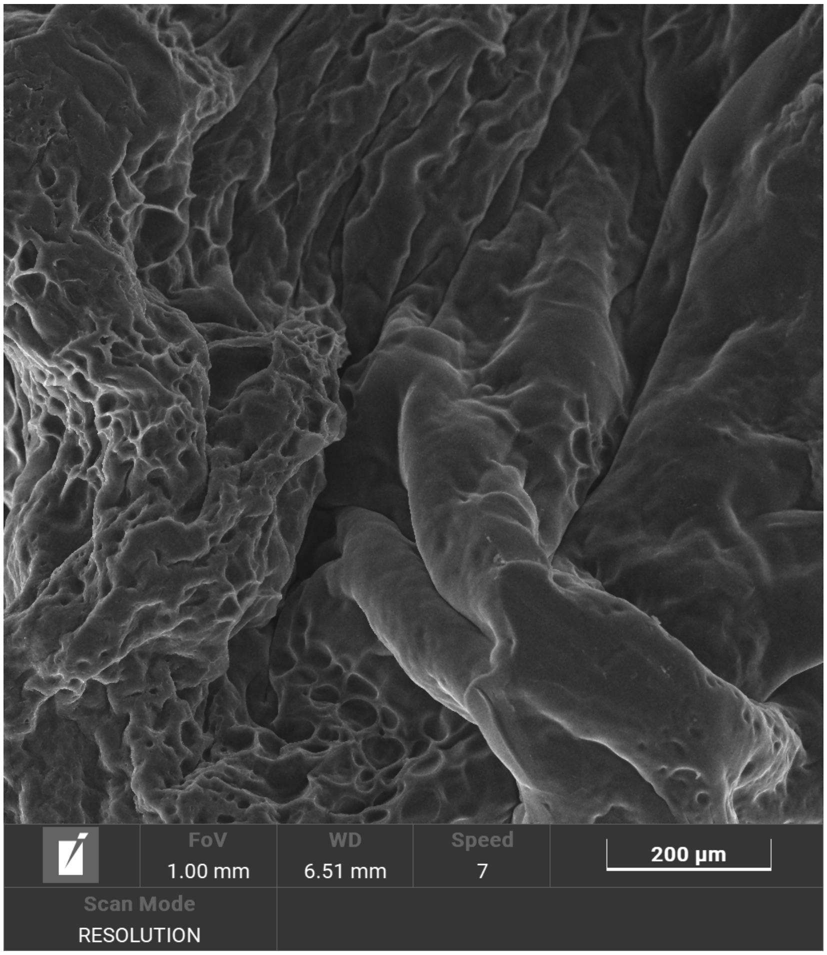

Figure S18: High-resolution SEM image of sample G6-P1 photopolymerized at 60°C, shown in the main text as Figure 10c.
